# Superior haplotypes of key drought-responsive genes reveal opportunities for the development of climate-resilient rice varieties

**DOI:** 10.1038/s42003-024-05769-7

**Published:** 2024-01-12

**Authors:** Preeti Singh, Krishna T. Sundaram, Vishnu Prasanth Vinukonda, Challa Venkateshwarlu, Pronob J. Paul, Bandana Pahi, Anoop Gurjar, Uma Maheshwar Singh, Sanjay Kalia, Arvind Kumar, Vikas K. Singh, Pallavi Sinha

**Affiliations:** 1 International Rice Research Institute (IRRI), South-Asia Hub, Hyderabad, India; 2 International Rice Research Institute, South Asia Regional Centre (ISARC), Varanasi, India; 3 Department of Biotechnology, CGO Complex, Lodhi Road, New Delhi, India; 4 Present Address: International Crops Research Institute for the Semi-Arid Tropics, Hyderabad, India

**Keywords:** Plant breeding, Plant physiology

## Abstract

Haplotype-based breeding is an emerging and innovative concept that enables the development of designer crop varieties by exploiting and exploring superior alleles/haplotypes among target genes to create new traits in breeding programs. In this regard, whole-genome re-sequencing of 399 genotypes (landraces and breeding lines) from the 3000 rice genomes panel (3K-RG) is mined to identify the superior haplotypes for 95 drought-responsive candidate genes. Candidate gene-based association analysis reveals 69 marker-trait associations (MTAs) in 16 genes for single plant yield (SPY) under drought stress. Haplo-pheno analysis of these 16 genes identifies superior haplotypes for seven genes associated with the higher SPY under drought stress. Our study reveals that the performance of lines possessing superior haplotypes is significantly higher (p ≤ 0.05) as measured by single plant yield (SPY), for the *OsGSK1*-H4, *OsDSR2-*H3, *OsDIL1-*H22*, OsDREB1C*-H3, *ASR3-*H88, *DSM3-*H4 and *ZFP182*-H4 genes as compared to lines without the superior haplotypes. The validation results indicate that a superior haplotype for the DREB transcription factor (*OsDREB1C*) is present in all the drought-tolerant rice varieties, while it was notably absent in all susceptible varieties. These lines carrying the superior haplotypes can be used as potential donors in haplotype-based breeding to develop high-yielding drought-tolerant rice varieties.

## Abstract (web summary)

Using a subset of 399 genotypes from the 3000 rice genomes panel, genotypes carrying superior haplotypes were identified for five genes associated with yield under drought stress that hold potential for the development of high-yield drought-tolerant varieties in rice.

## Introduction

Rice is a crucial food crop that sustains over half of the world’s population. However, global climate change is expected to lead to a 51% reduction in rice cultivation and production in the next century. In South and Southeast Asia, drought is a common problem during the growing season, particularly in rainfed environments. This affects around 23 million hectares of rice production areas in Asia^1^. Drought stress can harm different stages of rice growth, but it is particularly harmful during the vegetative and reproductive stages, resulting in a significant decline in grain yield^2^. Grain yield per plant (SPY), a complex quantitative trait, is regulated by polygenes. It is directly impacted by yield-component traits like panicle number, seed weight, and seed number. Additionally, yield-related traits such as biomass, harvest index, plant architecture, adaptation, and resistance to biotic and abiotic stresses can indirectly influence grain yield^3^. However, due to the constant drought conditions, unstable rice production has severe social and economic impacts. Developing rice varieties with better grain yield and quality under both non-stress and drought-stress conditions is necessary to mitigate the challenges caused by drought.

To address the above challenges, several major and minor QTLs associated with grain yield under drought stress conditions (*qDTYs*) have been identified in rice such as *qDTY*_*1*.*1*_^4,5^*, qDTY*_*2.1*_^6^, *qDTY*_*2.2*_^7^, *qDTY*_*2.3*_ and *qDTY*_*12*.*1*_^8^*, qDTY*_*3.1*_ and *qDTY*_*6.1*_^9,10^, *qDTY*_*4.1*_, *qDTY*_*9.1*_ and *qDTY*_*10.1*_^11^. A research study found that 16 drought grain yield QTLs were present across all chromosomes with the exception of 5, 7, and 8^12^. However, a meta-QTL analysis identified 14 MQTLs on seven chromosomes (1, 2, 3, 4, 8, 10, and 12) with a 4–28% effect on drought grain yield^13^. Introgression of prominent QTLs (*qDTY*_*1.1*_, *qDTY*_*2.1*_, *qDTY*_*2.2*_*, qDTY*_*3.1*_, *qDTY*_*3.2*_*, qDTY*_*6.1*_, and *qDTY*_*12.1*_) into high-yielding but drought-susceptible mega varieties including IR64, MTU1010, TDK1-*Sub1*, Savitri, Swarna-*Sub1*, Samba Mahsuri, and Vandana imparted better yield advantage and consistent effects in multiple environments under drought stress in different genetic backgrounds^14–18^. Marker-aided *qDTY* pyramiding in Indian elite rice varieties (Sahbhagi dhan, DRR Dhan 42, CR Dhan 801, Naveen, and PB 44) showed significantly superior performance under reproductive stage drought stress conditions^19–23^. Improved cultivars with either single or different combinations of *qDTYs* have already been released in many countries^16^.

Recent advancements in high-throughput genotyping technologies have greatly improved the process of crop trait mapping through NGS-based methods such as QTL-seq, MutMap, Indel-Seq, or BSR-Seq^24–27^. This has led to the faster development of new crop varieties. Genome-wide association studies (GWAS) have effectively identified favorable alleles associated with important agronomic traits in crops such as rice^28,29^. Whole-genome sequencing of diverse accessions has also made it easier to identify significant marker-trait associations, QTLs, QTNs, candidate genes, and superior haplotypes for targeted traits. A new approach called haplotype-assisted forward/backward breeding also has emerged, where superior haplotypes are identified and combined to create tailor-made varieties for crop improvement programs^30–32^. A haplotype refers to a set of closely linked DNA variations within a gene that tend to be inherited together. Earlier, haplotype and phenotype (*haplo-pheno)* analysis was utilized to explore the relationship between haplotypes and phenotypic traits. If the average phenotypic performance of the group of individuals with a particular haplotype is significantly higher than those with other haplotypic groups, it is considered as the superior haplotype^33^. In rice, superior haplotypes have been identified for traits such as grain yield and quality, low glycaemic index, and deep-water rice^30,32,34^. Similarly, the *haplo-pheno* analysis identified superior haplotypes for three genes present in 17 genotypes of 292 pigeon pea reference set associated with drought component traits^33^. This approach can broaden the existing genetic pool for developing high-yielding, climate-smart crop varieties.

This study aimed to determine the genes and superior haplotypes associated with the single plant yield trait in rice under drought stress. A haplotype diversity analysis was conducted on 95 genes that affect drought tolerance in rice, using a panel of 3K-RG genotypes. Furthermore, a candidate gene-based association study was conducted on a panel of 399 genotypes, selected from the 3K-RG panel, to investigate the relationship between these genes and drought tolerance.

## Results

### Haplotype analysis of 95 drought-responsive genes across the 3K-RG panel

**Fig. 1:**
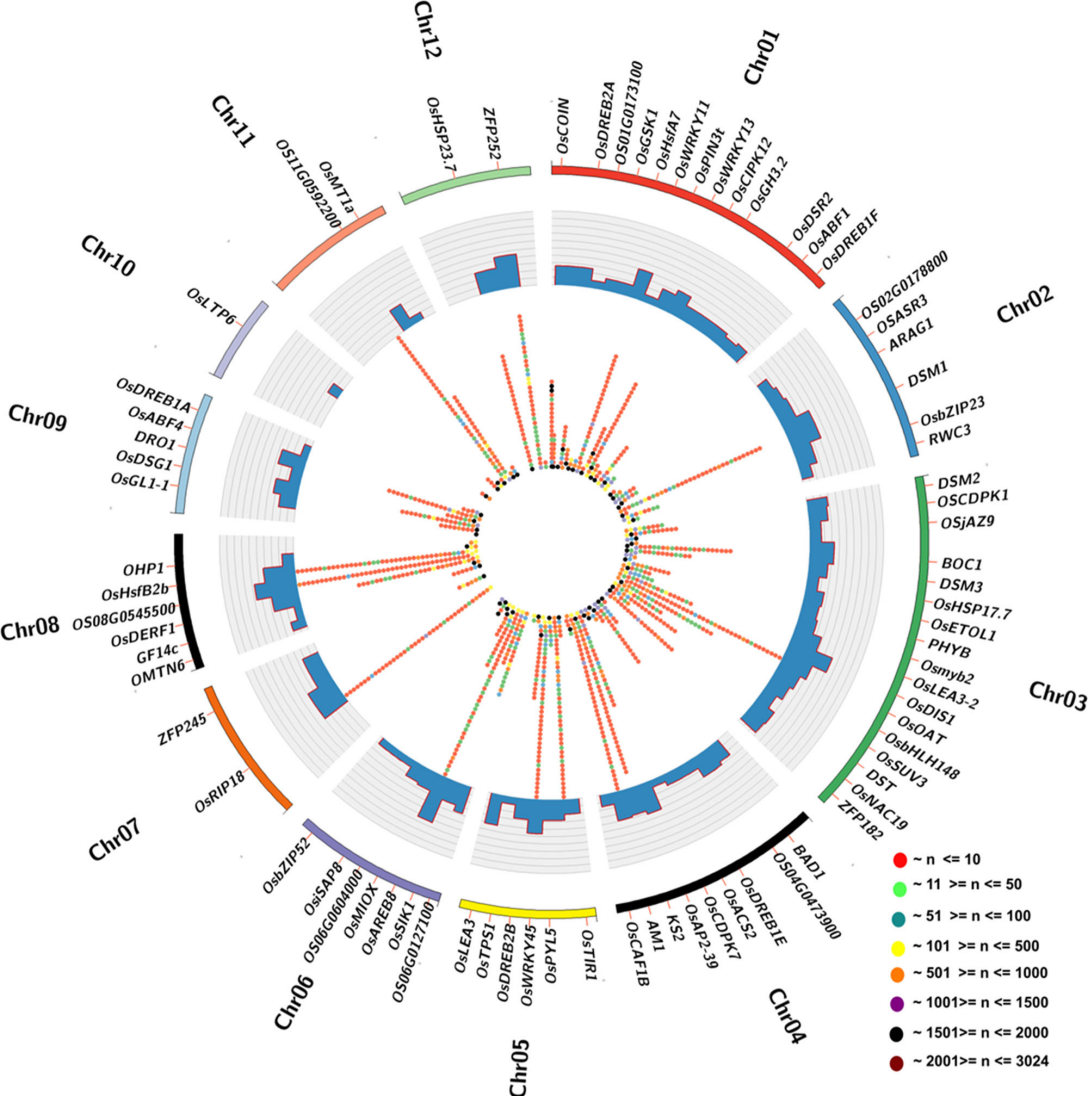
Haplotype diversity in the drought-responsive genes. The frequency and relative distribution of drought-responsive genes carrying >2 haplotypes in subset panel of 3K rice genomes are represented in the Circos diagram. The outermost circle represents 12 rice chromosomes (Chr 01–Chr 12) in different colors, and the inner two circles represent the number of haplotypes and their frequency distribution. The histogram displays the number of haplotypes obtained for each gene. The bubbles represent the frequencies of each haplotype particular to the gene. The range of haplotype frequencies is represented by different colors as indicated.

A haplotype analysis was conducted on 95 genes known to play a role in drought tolerance, using a panel of 3K-RG (Supplementary Data 1). These genes were chosen from various mapping studies and omics approaches in the public domain. The analysis revealed 2158 haplotypes, ranging from 1 to 274 haplotypes per gene. It was found that *OsHSP17.0*, *OsMTN4*, *OsDREB1F*, *OsDHODH1*, *OsDSG1*,*OsCAF1G*, and *wdl1* have only one haplotype each, while the *OsAbF2/OsAREB8* gene had the highest diversity with 274 haplotypes (Supplementary Data 1). The frequency and distribution of genes >2 haplotypes in the subset panel of 3K-RG are shown in Fig. 1. Each gene’s haplotype was analyzed to identify structural variations in the coding regions and their potential relationship to phenotypic traits.

### Phenotyping of the subset panel

To study the impact of drought on the yield of individual plants, a subset of 399 genotypes from the 3K-RG panel was selected to represent a wide range of diversity (Supplementary Data 2). This subset was then evaluated for yield under drought conditions in two crop seasons [Dry Season (DS) 2019 and Dry Season (DS) 2020] (Supplementary Fig. 1). The analysis using restricted maximum likelihood (REML) revealed a significant difference in yield among genotypes (*σ*^*2*^*g*) under both seasons and conditions (Supplementary Table 1). As expected, the average yield of the subset under drought conditions was lower than that under normal conditions in both seasons.

The SPY of 399 genotypes under stress conditions ranged from 0.6 to 16.8 g with higher variability than normal conditions (4.0–35.8 g). This variability was more significant under stress conditions (GCV 40.39% in DS, 2019 and 51.25% in DS, 2020) than under normal conditions (21.87% GCV in DS, 2019 and 18.83% GCV in DS, 2020). Repeatability for SPY was high (over 60%) in both normal and stress conditions, with a range of 67.62–77.52% and 76.245–77.17%, respectively. The percentage of the genetic advance of means (GAM) was also high in all conditions and seasons, but more so under stress conditions.

### GWAS identifies candidate genes associated with yield under drought stress

Candidate gene-based GWAS (c-GWAS) was conducted to identify genetic markers associated with yield under drought stress in rice. A total of 0.6 million SNPs in 95 genes were analyzed, leading to the identification of 69 Marker Trait Associations (MTAs) associated with yield (Supplementary Fig. 2 and Supplementary Data 3). These MTAs were distributed across all the chromosomes, except 4, 5, and 7, and explained between 2.00% and 4.48% of the phenotypic variation. The highest number of MTAs was found on chromosome 1 (25), while the lowest number was found on chromosomes 6, 9, and 10 (1 each). The candidate genes located in the vicinity of the significant signal were analyzed for known molecular functions, and several of them, such as *OsDREB1C*, *OsDREB1F*, *OsNAC10*, and *ZFP182* were responsible for specific DNA-binding transcription factor activity. These transcription factors are important in controlling the expression of a variety of genes associated with drought tolerance. Additionally, other genes were found to be involved in binding processes and catalytic activities, such as *OsGSK1*, *OsMT1a*, *OsDIL1,* and *OsHSP23.7* (Supplementary Fig. 3).

Expression patterns of the targeted genes revealed that these genes were prominently expressed in reproductive tissues (Supplementary Fig. 4a, b). In particular, *OsGSK1*, *OsDSR2*, *OsNAC10*, *GF14c*, and *OsHSP23.7* displayed high expression levels in the spikelet and panicle. Moreover, *ASR3*, *OsSRO1c*, *OsNAC10*, and *OsMT1a* had an expression pattern that was consistent across the developmental stages but was enhanced during the reproductive stage, suggesting they may have a vital role in drought stress responses. Additionally, a co-expression network of the selected genes gave insight into the functional aspect of genes that are positively co-expressed. The top 10 co-expressed were identified with Pearson’s correlation coefficient and visualized on circular plots (Supplementary Fig. 5). For *OsDSG1*, genes positively co-expressed were zinc finger family proteins and WD40-repeat family proteins (Supplementary Fig. 5a) while in the case of *OsSRO1c*, most of the co-expressed genes are transcription factors (Supplementary Fig. 5b). *OsWAK89b* and NAC transcription factor was found to be co-expressed with *OsDERF1* (Supplementary Fig. 5c). Similarly, *OsDSR2* was found to be co-expressed with kinesin motor-containing protein (Supplementary Fig. 5d).

### Superior haplotypes of associated genes for drought responsiveness

The haplotype analysis across the 16 associated genes uncovered a wide range of haplotype diversity. The number of haplotypes identified varied significantly, with *OsDREB1F* exhibiting the lowest number of haplotypes (1), while *ASR3* displayed the highest number of haplotypes (91). The major haplotypes were highly prevalent, with some, including *OsDREB1F*-H1, exhibiting a frequency as high as 100%. This indicates that major haplotypes are likely to be common in the population and may play an important role in determining the genetic makeup of the organisms. On the other hand, the minor haplotypes were found to have a low frequency, with some as low as 0.75% (*ASR3-*H88) (Supplementary Data 4). These haplotypes are likely to be rare in the population and have a significant impact during reproductive stage drought stress tolerance.

**Fig. 2:**
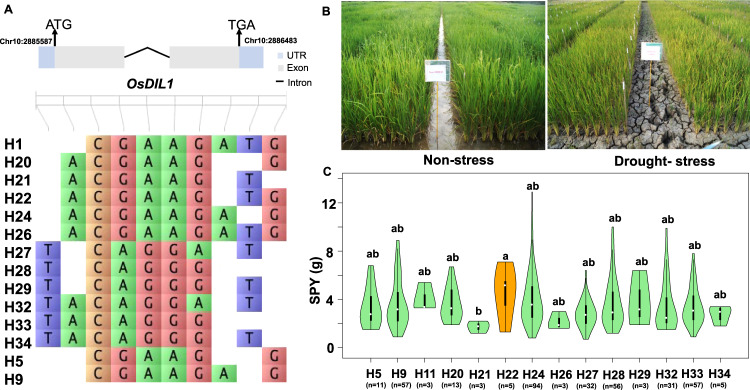
Haplotype analysis of the *OsDIL1* gene associated with the trait across the subset panel. **A** Haplotypic variation in the *OsDIL1* gene associated with single plant yield trait (SPY) in the subset panel. **B** Phenotypic evaluation of the subset panel (*n* = 399) in field conditions under non-stress and drought stress conditions. **C** Violin plot showing variation in SPY trait in rice accessions significant at *p*-value < 0.05. Different alphabets denote significant differences between haplotypes. The median is depicted by the horizontal line in the box. Note: Haplotype variation reflects only those haplotypes that are used in haplo-pheno analyses. The violin plot uses an orange color to depict the distribution of the superior haplotype, while the green color is employed to represent the distribution of the remaining haplotypes associated with the *OsDIL1* gene.

Haplo-pheno analysis was performed to identify the genes responsible for yield under drought stress by comparing the average yield of groups with different haplotypes. We identified superior haplotypes for only seven out of the 16 associated genes. For instance, among the 14 haplotypes identified for *OsDIL1*, H22 was identified as the superior haplotype (Fig. 2). Similarly, for the genes *OsGSK1*-H4, *OsDSR2*-H3, *ASR3*-H88, *DSM3*-H4, *ZFP182*-H4, and *OsDREB1C*-H3 were determined to be the most favorable haplotypes (Supplementary Fig. 6). Notably, we found that lines possessing superior haplotypes exhibited a significantly higher yield (8.5–62.5%) as compared to lines without superior haplotypes (Table 1).

**Table 1:** Average performance in terms of single plant yield (g) of the genotypes possessing superior haplotypes in comparison to another group of haplotype.

Gene	*Os_ID*	Total no. of haplotypes	SH	Average performance of individuals with SH	Range of average performance of haplotypic groups other than SH group	Top performing three donors possessing SH
*OsGSK1*	*Os01g0205700*	4	**H4**	H4-4.2 g^a^	H3-3.2 g^b^ - H2-3.9 g^a^	Da Nuo IH Pen Shim Ming Deng Deng Qi
*OsDSR2*	*Os01g0839200*	3	**H3**	H3-3.8 g^a^	H1-3.1 g^b^ - H2-3.4 g^a,b^	Lalka (lal dhan) Da Nuo India dular
*OsDREB1C*	*Os06g0127100*	6	**H3**	H3-5.2 g^a^	H5-2.9 g^b^ - H6-3.1g^b^	Chandina Hodarawala Merle
*ASR3*	*Os02g0543000*	32	**H88**	H88-6.5 g^a^	H99-2.7 g^bc^ - H130-5.2^ab^	Lal taura Aus 359 Sada aus
*DSM3*	*Os03g0230500*	4	**H4**	H4-5.5 g^a^	H2-3.3 g^b^ - H5-4.2 g^ab^	Chandina L10833 IR28
*ZFP182*	*Os03g0820300*	16	**H4**	H4- 4.7 g^a^	H14-2.6 g^ab^ - H8-4.0 g^ab^	ARC 18533 Salsi Napdai
*OsDIL1*	*Os10g0148000*	14	**H22**	H22- 4.5 g^a^	H21-1.7 g^b^ - H9-4.0^ab^	T. kaug pao Nam Sa-Gui 19 E Zi 96

Duncan’s analysis was employed to test statistical significance at *P* < 0.05. Means followed by different letter for each haplotype groups are significantly differenct. *Haplo-pheno* analysis of only those haplotype groups was performed in which at least three genotypes were present. *SH* Superior haplotype.

### Identification of genotypes carrying superior haplotypes

**Fig. 3:**
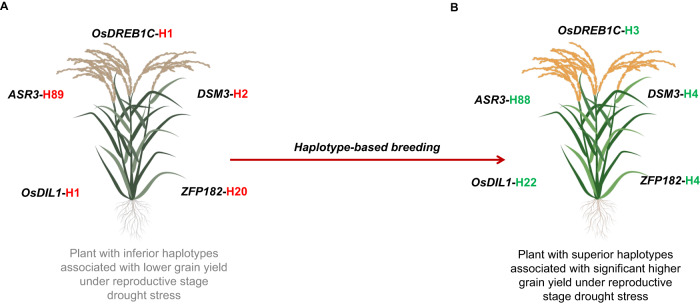
Tailor-made rice with superior haplotypes for developing high-yielding drought-tolerant varieties. **A** Representative rice plant carrying inferior haplotype combination (*OsDREB1C-H1*, *ASR3-H89*, *OsDIL1*-H1, *DSM3*-H2, and *ZFP182*-H20). **B** Superior haplotype combination (*OsDREB1C-H3*, *ASR3-H88*, *OsDIL1*-H22, *DSM3*-H4, and *ZFP182*-H4) for SPY trait under reproductive stage drought stress. New breeding lines can be developed by introgressing superior haplotype combinations through haplotype-based breeding. SPY single plant yield.

Our discovery of superior haplotypes has enabled us to identify the most promising genotypes from a subset panel, which can be utilized in a haplotype-based breeding program to design drought-tolerant plants. Across all genotypes with superior haplotypes, the average plant yield ranged from 3.8 to 6.5 g (Table 1). In order to assess how genetic variation can influence the response of a subpopulation to drought stress we have compared haplotype frequencies across subpopulations. Haplotype frequency of the 7 associated genes showed substantial differences among the subpopulation (Supplementary Data 5). We found that the superior haplotype for the associated genes was present only in the indica and aus subpopulations, while none of the superior haplotypes was present in the Japonica subpopulation (Supplementary Table 2). This result further affirms that indica varieties are better adapted to survive in drought conditions. Identifying novel haplotypes can provide important insight into the genetics of the sub-population and can help researchers gain a better understanding of evolutionary processes. In addition, we have identified accessions that carry multiple superior haplotypes, which would significantly expedite the process of customizing rice development for yield under drought stress. Notably, we have identified genotypes with two distinct combinations of superior haplotypes for genes, which result in different phenotypes depending on the specific combination of superior haplotypes utilized. For example, the DONDRADAO: *IRIS_313-11411* genotype from Brazil and CHANDINA: *IRIS_313-9917* from Sri Lanka have superior haplotypes for two gene combinations for the SPY trait: *OsDREB1C*-H3, *ZFP182*-H4, and *DSM3*-H4 (Supplementary Table 3). The combinations of identified superior haplotypes governing the foremost drought component trait SPY in the current study could be deployed to develop drought-tolerant rice cultivars through a haplotype-based breeding approach (Fig. 3).

### Validation of superior haplotypes in drought-tolerant rice varieties

**Fig. 4:**
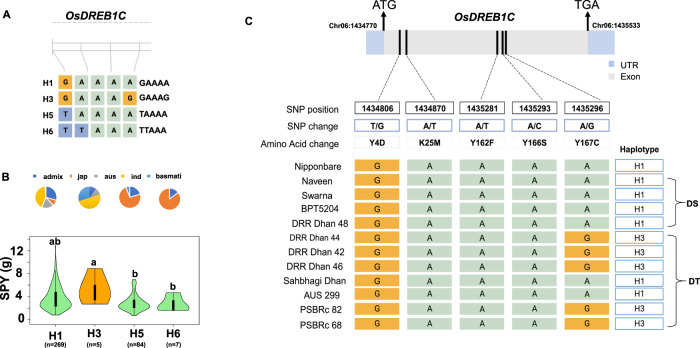
Validation of the superior haplotypes in drought-tolerant rice varieties for the *OsDREB1C* gene. **A** Haplotypic variation of *OsDREB1C* gene in 3K-subset. **B** Subpopulation- wise distribution of haplotypes in the subset and violin plot showing variation in SPY trait in rice accessions significant at p-value < 0.05. The violin plot uses an orange color to depict the distribution of the superior haplotype, while the green color is employed to represent the distribution of the remaining haplotypes associated with the *OsDREB1C* gene. **C** Gene structure and haplotype sequence variation of the *OsDREB1C* gene. Four drought-susceptible (DS) and seven drought-tolerant (DT) varieties of rice were examined for haplotypic variation between the superior/favorable (H3) and the unfavorable (H1) haplotypes. DT drought tolerant lines, DS drought susceptible lines. Figure created using Biorender (https://biorender.com/ accessed on 16 August 2023).

The validation of superior haplotypes was performed by conducting whole genome re-sequencing (WGRS) using the Nova Seq Illumina platform on eight known rice varieties (four drought-tolerant and four drought-susceptible). The average depth of coverage ranged from 16-fold to 45-fold, with a mean genome coverage of 91% (as depicted in Supplementary Table 4). Through our analysis, we compared the superior/favorable haplotypes to the inferior/unfavorable haplotypes along with drought-tolerant and susceptible varieties of the five identified genes. However, for two of the seven genes, *OsGSK1* and *OsDSR2*, the superior haplotypes were present in a large proportion of the genotypes (47% and 13%, respectively), so we did not consider them for further analysis and performed validation on only five genes (Table 2). Interestingly, for the *OsDREB1C* gene, the reported superior haplotype (H3 with the haplotype sequence—GAAAG), was present in all the resistant varieties (DRR Dhan 42, DRRDhan 44, DRR Dhan 46, PSBRc 68, and PSBRc 82) except Sahbhagi Dhan and Aus 299. Conversely, the unfavorable haplotype (H1 with the haplotype sequence—GAAAA) was observed in all drought-susceptible varieties, and it was linked to reduced yield under reproductive stage drought stress. This superior haplotype was observed to be absent in drought-susceptible rice varieties but was selectively bred in drought-tolerant varieties through domestication and modern breeding practices. The haplotypic differences for the *OsDREB1C* gene are presented in Fig. 4. For the other four genes, we did not find any correlation between resistant and susceptible varieties.

**Table 2:** Validation of superior haplotypes on known drought-tolerant and susceptible varieties.

Gene	Chromosome	Total no. of haplotypes	Superior haplotype	Accessions with SH	Superior haplotype sequence	Drought-tolerant varieties							Drought-susceptible varieties			
						DRR Dhan 44	DRR Dhan 42	DRR Dhan 46	Sahbhagi Dhan	PSBRc 82	PSBRc 68	Aus 299	Naveen	Swarna	DRR Dhan 48	BPT5204
*OsDREB1C*	6	4	H3	5	GAAAG	GAAAG (H3)	GAAAG (H3)	GAAAG (H3)	GAAAA (H1)	GAAAG (H3)	GAAAG (H3)	^a^GAAAA (H1)	^a^GAAAA (H1)	^a^GAAAA (H1)	^a^GAAAA (H1)	^a^GAAAA (H1)
*ASR3*	2	32	H88	3	T–A--TA------T–A	^b^T–A--TA–C------A	^b^T–A--TA-C------A	^b^T–A--TA–C------A	^b^T–A--TA–C------A	^a^T-A--TA---G--TT (H99)	^b^T–A--TA–CGG–ATT	^a^T–A--TA--GG—TT (H112)	^b^T–A--TA–C------A	^b^T–A--TA–C------A	^b^T–A--TA–C------A	^b^T–A--TA–C------A
*DSM3*	3	4	H4	3	GGGAAAGT	^a^GATAAAAT (H2)	^a^GATAAAAT (H2)	^a^GATAAAAT (H2)	^a^GATAAAAT (H2)	^b^A/GT/GAATACA/G	^a^GGGAAACGT (H3)	^a^GGGAAACGT (H3)	^a^GATAAAAT (H2)	^a^GATAAAAT (H2)	^a^GATAAAAT (H2)	^a^GATAAAAT (H2)
*ZFP182*	3	16	H4	10	A----A–C CGG--T	^b^A----A–C–CGGT–T	^b^A----A–C–CGGT–T	^b^A----A–C–CGGT–T	^b^A----A–C–CGGT–T	^a^A-TA-A-C-CGG—T (H8)	^a^A-----A-C-C----– (H1)	A----A–C–C–G—T (H2)	^b^A----A–C–CGGT–T	^b^A----A–C–CGGT–T	^b^A----A–C–CGGT–T	^b^A----A–C–CGGT–T
*OsDIL1*	10	14	H22	3	T–ACGAAG–TGC	^b^TTACGAAGAT–C	^b^TTACGAAGAT–C	^b^TTACGAAGAT-C	^b^TTACGAAGAT-C	^a^T-ACGAAGA-GC (H24)	^a^T-ACGAAGA-GC (H24)	^a^TT–CAGGG---C (H24)	^b^TTACGAAGAT–C	^b^TTACGAAGAT–C	^b^TTACGAAGAT–C	^b^TTACGAAGAT–C

^a^These genotypes possess other alternate haplotypes than the superior haplotype identified for the gene of interest.

^b^Haplotype not present in the subset panel.

### In silico structural analysis of the candidate genes

The impacts of nonsynonymous single nucleotide polymorphisms (nsSNPs) were assessed in order to study the structural changes in the candidate genes. SIFT scores were used to evaluate the effects of the nsSNPs on protein structure for each gene. As a result, only one SNP at position 1434870 (A/T) in the *OsDREB1C* gene was discovered to have a functional mutation. When analyzing the protein structure, this specific mutation with a SIFT score of 0.00 caused the replacement of methionine for lysine at the 25th amino acid position resulting in a deleterious effect. The remaining four missense mutations, on the other hand, were not found to result in any substantial structural alterations and were deemed tolerable (Supplementary Data 6). The superimposed model of the translated protein sequences representing the unfavorable (H1) and favorable haplotypes(H3), as predicted by SWISS-MODEL, indicates that the mutation has no significant impact on the protein structure. Further, in order to select the superior haplotype for the *OsDREB1C* gene, KASP (kompetitive allele-specific PCR) primer has been designed. The details of the primer are supplied in Supplementary Data 7 for easy inclusion into breeding programs, enabling its efficient use. Similarly, for other genes, we could not find any deleterious effect nsSNPs.

## Discussion

Haplotype-based breeding has the potential to solve issues in modern agriculture by using superior alleles from target genes to create new traits. Genetic variation in the diversity panels can significantly increase genetic gain in this integrative pre-breeding approach. Haplotype analysis of key genes to identify trait-associated variations revealed superior haplotypes for heat stress and fertility, grain cooking, and eating quality^35,36^. In addition, sequencing-based mapping strategies can also help us to identify superior haplotypes for different traits to facilitate tailor-made crop plants *via* haplotype-based breeding^30^.

Although the successful cloning of functional genes in rice stands as a notable achievement, the challenge lies in the slow translation of these discoveries into concrete progress in molecular breeding. A substantial challenge exists in accessing the original donors for integration into these programs. The primary objective of this study is to narrow this gap by identifying superior or alternative haplotypes suitable for integration into molecular breeding programs. The main focus of our current study employing c-GWAS is to specifically uncover superior haplotypes of already cloned and functionally characterized genes reported in the literature through various studies (GWAS, overexpression/silencing, or omics experiments). We shortlisted 95 major drought tolerance genes from the RiceFun database, to determine the genes and superior haplotypes associated with the single plant yield trait in rice under drought stress.

The present study analyzed the haplotype diversity of 95 key drought-responsive genes in 399 diverse genotypes from the 3K-RG panel. Results showed significant variation among the genotypes for the SPY across the environments, indicating a high level of segregation. The study also found that drought stress has a major influence on creating variation in the genotypes and that the degree of drought tolerance differs among the accessions. Repeatability estimates were high for all seasons and similar in stress and non-stress environments, suggesting that similar results would be obtained if the trial will be repeated under similar conditions. Further, association studies were conducted to detect genetic variants and underlying candidate genes associated with the agronomically important trait SPY.

c-GWAS is a powerful approach that uses the correlation between genetic variants and trait differences based on linkage disequilibrium (LD) to study the genetic mechanism of complex traits^37,38^. We studied the association of SPY, which is known to be regulated by various indirect and direct traits with drought stress conditions^39,40^. Our study identified 69 strongly associated MTAs in 16 candidate genes with significant Phenotypic Variance Explained (PVE) for SPY under reproductive stage drought conditions on chromosomes 1, 2, 3, 6, 8, 9,10, 11, and 12. GO annotation revealed that the 16 genes identified involve different molecular functions such as sequence-specific DNA-binding transcription factor activity, protein binding, catalytic activity, kinase activity, nucleic acid binding, and lipid-binding activity (Supplementary Fig. 3). These genes have been reported to play a role in processes such as floral development, stress signaling transduction, grain yield under drought conditions, wax accumulation, and drought tolerance at the vegetative and reproductive stages^41–45^.

Out of the 69 MTAs identified, 25 showing the strongest association signal with 4.48% PVE were found in the 50-kb region of the *LOC_Os01g10840* gene on chromosome 1, which encodes a glycogen synthase kinase gene involved in abiotic stress tolerance. 16 MTAs were present within the 50-kb flanking region of the *LOC_Os11g47809* gene, which belongs to the metallothionein-like protein family. There were 6 MTAs within the *LOC_Os3g60560* (*ZFP182*), a C_2_H_2_ type zinc finger protein that plays a critical role in abiotic stress tolerance (Supplementary Data 3). The MTAs identified in this study were also compared to previously identified QTLs using the QTL Annotation Rice Online (Q-TARO) database. Interestingly, two SNPs (S1_35970278 and S9_286465239) present within the candidate loci of *OsDSR2* and *OsDSG1* were found to be co-localized with previously identified major *qDTY* QTLs (*qDTY*_*1.1*_ and *qDTY*_*9.1*_)^4,46^, and three SNPs (S1_42747553; S6_182471138; S8_264245022) were found to be in the vicinity of *qDTY*_*1.1*_*, qDTY*_*6.3*_, and *qDTY*_*8.1*_, respectively^18,47^ (Supplementary Table 5). *OsDSR2* is a member of the *DUF966* gene family and negatively regulates brassinosteroid signaling, playing a role in stress signal-transduction pathways and flower development^48^. The *OsDSG1* gene encodes a U-box E3 ubiquitin ligase and positively regulates cell elongation or division in different organs^49^. The colocalized MTAs identified in our study with the previously reported QTLs and the superior haplotypes of the candidate genes might prove helpful in future rice breeding programs for increasing grain yield under drought conditions. Furthermore, c-GWAS analysis using the drought susceptibility index (DSI) data provided further support to our findings, as our observations revealed a significant overlap between the identified MTAs and the genes associated with drought tolerance. This remarkable overlap reinforces the consistency of our results and is in line with the findings obtained from GWAS analysis using SPY data (Supplementary Data 8). These results highlight the consistency and significance of these genes in relation to drought tolerance, highlighting their potential to enhance plant productivity under challenging conditions.

In c-GWAS analysis, out of 69 MTAs, only three MTAs were found to be falling directly within the genic regions (*OsSRO1c*, *GF14c*, and *OsMT1a*). To explore the involvement of other genes along with our candidate genes we explored all the genes present in the 50 kb region other than the cloned and functionally characterized candidate genes that we have used in the present study. As a result, 302 genes were found in the vicinity of the region. Out of these 302, only 32 genes (16 previously identified in the present study and 16 additional genes reported in the new analysis) were cloned or functionally characterized based on the *Ricefun* database. *Haplo-pheno analysis* of 16 additional new genes revealed that only two genes, *ZFP15* (H3) and *OsPRP3* (H14), exhibited superior haplotypes. *ZFP15*, is a C_2_H_2_-type zinc finger protein and plays a significant regulatory role in spike development. However, it is not associated with abiotic stress responses as previously reported^50^. *OsPRP3* encodes for flower-specific Proline-rich protein (PRP), is involved in cell wall assembly during flower maturation, and has also been shown to contribute to cold stress tolerance in rice^51^. However, for most of these genes, there was no variation observed in the 3K-RG panel, as indicated in Supplementary Table 6. Our findings provide strong support and validation, underscoring the significance of the identified genes with superior haplotypes in shaping the genetic landscape of drought stress response in rice. These insights shed light on the potential role of these associated genes and underscore their importance in future breeding and improvement programs.

Scanning gene expression profiles at different development stages helps to establish the link between a gene’s expression and its biological function, including its role in variations in phenotype^52^. Combining GWAS with co-expression analysis improves the validation of MTAs and understanding of the molecular framework regulating traits of interest^53^. We found that *OsHSP23.7* was highly expressed in spikelets and most of the developmental stages, of which stem elongation and mature grain stage were the two best matches, suggesting its critical role throughout the life cycle of the rice plant. *OsNAC10* was highly expressed in the root and at the dough and mature grain stage, suggesting that *OsNAC10* is involved in drought tolerance and high grain yield *via* root growth^43^. Interestingly, the NAC transcription factor gene (*LOC_Os11g03310*) and *OsWAK89b* (*LOC_Os09g38834*) were co-expressed with the *OsDERF1*. *OsWAK* has been previously reported to control panicle number and grain yield^54^. Similarly, *OsSRO1c* was found to be co-expressed with transcription factors such as C_2_H_2_, zinc finger protein, GRAM domain-containing protein, and NAC domain-containing proteins, which is consistent with its role as a transactivator. *OsDSG1* was also found to be co-expressed with zinc finger-like protein-encoding genes. The role of zinc fingers in regulating plant architecture and grain yield has already been established in rice and other crops^55^. Although co-expression profiling does not capture all functional interactions and further evidence is required to validate and understand the co-expression network, these data provide informative clues about genes contributing to the same biological process associated with grain yield. In the future, integrating genetic, genomic, transcriptomic, proteomic, metabolic, and phenotypic data through a systems biology approach will provide a more comprehensive understanding of the associations between genotype and phenotype in plants.

Phenotypic validation of 16 candidate genes associated with drought tolerance in a subset of 399 rice genotypes identified superior haplotypes for seven genes (*OsGSK1*, *OsDSR2, OsDREB1C*, *ASR3*, *DSM3*, *ZFP182*, and *OsDIL1*) for drought responsiveness. Among these genes, *OsGSK1* is a glycogen synthase kinase gene that improves tolerance to abiotic stress, while *OsDIL1* encodes a lipid transfer protein that aids in drought tolerance at vegetative and reproductive stages^41,45^. *OsDSR2*, a member of the SRO (SIMILAR TO *RCD* ONE) family acts as a negative regulator of BR-signaling and increases sensitivity to salt and drought stress when overexpressed^48^. *DSM3*, a member of the *OsITPK* family, plays a crucial role in stress response, and maintaining an optimal expression level is essential for enhancing drought and salt tolerance in rice^56^. *ASR3* on the other hand, is involved in maintaining higher photosynthetic activity during cold and drought stress and influencing plants’ hormone and sugar status, at various growth and developmental stages during the plant life cycle^57^. *OsDREB1C* regulates photosynthesis and nitrogen utilization in response to drought stress, while *ZFP182*, a C_2_H_2_ type of zinc finger protein, promotes multiple stress tolerance by regulating ABA-induced antioxidant defense response in plants^55,58^.

To further validate our findings and gain insight into the genetic factors contributing to drought tolerance in rice, we conducted whole-genome re-sequencing on four drought-tolerant and four drought-susceptible released varieties. Our analysis focused on non-synonymous SNPs and InDels within the 5’UTR, CDS, and 3’UTR regions in the five identified genes.’ By comparing the most favorable and unfavorable haplotypes across drought-tolerant and susceptible varieties, we sought to identify the association of superior haplotypes with higher yield under reproductive stage drought stress. Additionally, we conducted a literature search to identify potential donors that have been previously utilized in conventional breeding and QTL mapping studies^12^.

The results revealed the consistent presence of superior/favorable haplotype H3 of the *OsDREB1C* gene in all drought-tolerant varieties except in Sahbhagi dhan and Aus 299, which possessed the H1 haplotype. However while looking into the parentage information, we noted that each drought-tolerant variety exhibited different immediate parentage and lacked shared lineage, suggesting potential genetic differences among them (Supplementary Table 7). However, the common superior haplotype (H3) present in the five selected genotypes reflected that there may have been some common great-grand parentage during the course of the development of these lines. This uniformity in haplotype distribution suggests that this specific haplotype could be a key determinant of drought tolerance in these varieties. The genotypes Sahbhagi dhan and Aus 299 were found to lack the superior haplotype of the *OsDREB1C* gene (H3) reflecting the presence of some other superior haplotype of any other genes not identified in the present study. This suggests that donors of *OsDREB1C* might have been used in previous drought tolerance breeding programs for improving drought tolerance and enhancing grain yield in these modern varieties. This finding aligns with recent research highlighting the crucial role of the *OsDREB1C* gene in increasing grain yield by up to 40% while reducing the growth duration time in rice^58^.

Interestingly we have not found any superior haplotypes of the other four genes *ASR3*, *DSM3*, *ZFP182*, and *OsDIL1* in the selected seven drought-tolerant lines and as expected in the susceptible lines. The genes and their superior haplotypes identified in the present study are novel and based on our validation results, it was noted that these genes are never being utilized in the drought breeding programs. This further reflects the importance of the identification of novel superior haplotypes to be deployed in breeding programs to develop climate-resilient drought-tolerant varieties. We found that the genotype (Chandina) carrying superior haplotypes for *OsDREB1C* and *DSM3* gene could be a potentially important target that can be utilized in breeding programs to enhance grain yield under drought stress.

We have also noted that the number of accessions used for *haplo-pheno* analysis in certain cases (*DSM3* and *ASR3* genes) was limited to three individuals in the superior haplotype group. Although based on the statistical analysis minimum of three individuals are required to analyze the datasets. However, the presence of background effects from other loci can influence the results. Therefore, to ensure more reliable conclusions, it is imperative to define a group with >5 individuals with minimum variance within the same group should be cut-off to start the analysis. Such analysis in the future will effectively reduce background noise from other loci. Furthermore, these findings also emphasize that these genes are novel and have not been utilized in any breeding programs for developing drought-tolerant varieties, representing the untapped potential for enhancing the performance of these varieties. Therefore, incorporating these superior haplotypes into breeding programs holds great promise for further improving drought tolerance in rice cultivars. Genotypes carrying superior haplotypes for these genes displayed higher levels of drought tolerance, as evidenced by their ability to maintain higher yields in drought conditions compared to genotypes with unfavorable haplotypes.

Our finding highlights the critical role of five genes (as represented in Fig. 3) in conferring drought tolerance in rice plants. These identified haplotypes hold promise for continued exploration and prospective utilization in breeding programs aimed at strengthening drought tolerance in rice. However, further functional validation of these haplotypes is required to establish their suitability as potential targets for genome editing to enhance drought tolerance. Overall, our data provide valuable insights into the genetic basis of drought tolerance in rice and present a potential strategy for enhancing rice production in water-limited environments.

## Methods

### Plant materials

The study used a panel of 399 genotypes from the 3K-RG panel, representing very early (60–70 days), early (71–80 days), and mid-early (80–100 days) duration genotypes from various sub-population groups, including *basmati, aus, admix, indica*, and *japonica*. The panel was analyzed for haplotype diversity and covers most *O. sativa* groups collected from various geographical regions across 55 rice-growing countries worldwide (Supplementary Data 2).

### Haplotype analysis of drought-responsive genes

In our study, we implemented a comprehensive approach to identify genes associated with drought tolerance in rice. We first selected major genes known to be involved in drought tolerance from the RiceFun database^59^. Additionally, we conducted extensive literature mining to identify additional functionally characterized genes associated with drought responsiveness. As a result, a total of 95 genes were considered for haplotype analysis (Supplementary Data 1). A subset of 399 rice genotypes from the 3K-RG panel was used for the haplotype analysis of the 95 selected drought-responsive genes. For haplotype analysis, full-length sequences of the selected genes were downloaded from the 3K rice whole-genome database (https://snp-seek.irri.org/_download.zul) and aligned to the Nipponbare reference genome using the BWA tool^60^. After the alignment, variant calling was done using Genome Analysis Toolkit (GATK)^61^. The identified variants were later utilized for haplotype analysis using Haploview software^62^. The Circos tool was used to visualize the relative distribution of genes >2 haplotypes in the 3K subset^63^.

### Drought-tolerance phenotyping

Experiments were conducted at the International Rice Research Institute (IRRI) South Asia Hub, located in Hyderabad, India at a latitude of 17° 32′ and longitude of 78° 16′, and an altitude of 540 m above sea level. 30-day-old seedlings of genotypes from the 3K-RG panel were transplanted in the main field, spaced 20 × 15 cm apart, and fertilized with a dose of 120–60–40 (N:P:K) kg/ha during two crop seasons (DS, 2019 and DS, 2020). The plots were arranged in an augmented RCBD design with 10 checks, which consisted of both drought-tolerant and susceptible varieties. The drought-tolerant checks included Vandana, Dular, Sahbhagi Dhan, DRR dhan 44, CR Dhan 801, Bahuguni Dhan1, Bahuguni Dhan 2, and Sukha Dhan 2, while MTU1010 and IR64 served as susceptible checks.

An established protocol for drought phenotyping was used^64^. To ensure synchronization between drought stress and flowering, staggered sowing, and transplantation were implemented, creating a 10-day interval between the three sets to align their flowering times for effective drought stress imposition. Irrigation of the drought plots was stopped one month after transplantation, and soil moisture content was measured using perforated PVC pipes inserted 1.0 m into the soil. Flood irrigation and drainage were applied when IR 64 plants showed leaf-rolling symptoms. Standard agronomic practices were followed, and grain yield per plant was recorded from five randomly chosen plants in each plot.

### Candidate gene-based association analysis

Association analysis was performed using 95 selected genes to identify significant marker–trait associations (MTAs) in response to SPY. The LD decay was determined using 600K SNPs, and it was observed to occur at 50-kb with a threshold *r*^2^ value of 0.2. Candidate gene prediction was performed within 50-kb upstream and 50-kb downstream regions of the genes. A *Q*-matrix was derived from ADMIXTURE software with *K* = 5 and included in association analysis^65^. Candidate gene-based GWAS (c-GWAS) analysis was performed using the GAPIT R package with three statistical models: mixed linear model (MLM), compressed MLM model (CMLM), and multiple loci MLM (MLMM)^66–68^. MTAs that surpassed the *p*-value threshold of *p* ≤ 0.005 and were common in all the models were considered as significant SNPs associated with the trait. The resulting significantly associated candidate genes were further used for *haplo-pheno* analysis.

### Haplo-pheno analysis

To identify the robust donors having superior haplotypes of the key drought genes we have combined data of DS (dry season) of 2019 and 2020 for the best linear unbiased prediction (BLUP) analysis. The average BLUP values of SPY from both years were utilized for the haplo-pheno analysis.

Haplo-pheno analysis was performed to associate the identified haplotypes of the selected genes with superior drought tolerance phenotypes. Haplotypes present in less than three accessions and heterozygous SNPs were removed from the analysis. The genotypes were then categorized based on haplotype groups, and superior haplotypes were identified using the phenotypic data of the individuals in each haplotype group. Haplotype-wise means of the single plant yield data were compared to define superior haplotypes. Duncan’s multiple range test (DMRT) was used to test the statistical significance among the mean of haplotype groups using the Agricolae package in R^69^. Groups with different letters in the graphs indicated significant differences among the groups at a *p* < 0.05 level of significance.

### Whole genome resequencing (WGRS) and variant identification

To validate the identified superior haplotypes, a total of eight varieties, including four drought-tolerant (DRR Dhan 42, DRR Dhan 44, DRR Dhan 46, and Sahbhagi dhan) and four drought-susceptible (Naveen, BPT 5204, DRR Dhan 48 and Swarna) were sequenced. The sequencing was done using Illumina Nova-Seq 6000 with 150 bp × 2 paired-end reads at 25× coverage, generating an average of 5 GB data per sample. Reads containing adapter sequences or stretches of ambiguous bases and those with low-quality scores were removed from the raw data, and reads with a Phred quality score ≥30 were retained. FASTQC was used to check GC content and duplicate reads, and the results were collectively visualized using multiQC^70^. The reference-based sequence assembly was performed using the BWA-MEM tool with Platinum standard Nipponbare reference genome IRGSP1.0^71^. Duplicate reads were removed and sorted according to coordinates using Picard tools. Mapping quality was checked for mapping percentage and genome coverage using Qualimap^72^. GATK 4.2.6.1 was used to call variants with SNP depth ≥10, and a genotypic call rate of 90%, and the variant file was saved in the VCF format. The obtained VCF format file was used to extract genetic variants of the candidate genes with superior haplotypes. The sequence of each variety was extracted using the fasta alternate reference maker command in the GATK toolkit. The gene sequence of each variety was checked for multiple sequence alignment for better visualization of variants. The haplotypes for each of the varieties were extracted and visualized with flapjack 1.22.04.2^73^. SIFT (http://sift.jcvi.org/www/SIFT_seq_submit2.html) a web-based tool that predicts whether amino acid substitution affects protein function and structure based on sequence homology and the physical properties of amino acids was employed to discover functional mutations. The predicted SIFT score ranges from 0 to 1. The amino acid substitution is predicted to be damaging if the score is <0.05 and tolerated if the score is >0.05.

### Meta-analysis and co-expression network of the associated genes

The expression data of the selected genes at various anatomical and developmental stages were obtained from Genevestigator software^74^ and co-expression analysis was performed using the Affymetrix Rice Genome Array platform. A co-expression network was created using the condition search tools and the perturbations profile in Genevestigator. The top 10 positively co-expressed genes were displayed in a circular plot with a Pearson correlation coefficient (PCC) score.

### Statistics and reproducibility

Single plant yield (SPY) data were recorded over two crop seasons in 399 accessions. Matured panicles were collected, and the corresponding yield in grams was calculated for each accession in three biological replicates. For GWAS, MTAs that surpassed the *p*-value threshold of *p* ≤ 0.005 and were common in all three models were considered as significant SNPs associated with the trait. Duncan’s multiple range test (DMRT) was used to test the statistical significance among the mean of haplotype groups using the Agricolae package in R^70^. Groups with different letters in the graphs indicated significant differences among the groups at a *p* < 0.05 level of significance.

### Reporting summary

Further information on research design is available in the Nature Portfolio Reporting Summary linked to this article.

### Supplementary information

Supplementary Information

Description of Additional Supplementary Files

Supplementary Data 1-3 and 5-8

Supplementary Data 4

Supplementary Data 9

Reporting Summary

## Data Availability

The WGRS sequencing data generated in this study are available in the SRA public repository under the bioproject accession number PRJEB70241. Sequence data used in this article can be found under the following accession numbers: ERR12304009 (DRR Dhan 42), ERR12303753(DRR Dhan 44), ERR12305485 (DRR Dhan 46), ERR12305492(Naveen), ERR12305489 (Swarna), ERR12305486 (BPT 5204), ERR12305487 (DRR Dhan 48), and ERR12305488 (Sahbhagi Dhan). Data supporting the findings of this work are available within the paper and its Supplementary Information Files. The source data behind the graphs in different figures in the paper are provided in Supplementary Data 9. The genetic materials that support the findings of this study are available from the corresponding authors upon request.
