# Supplementary material for: Superior haplotypes of key drought-responsive genes reveal opportunities for the development of climate-resilient rice varieties
Source: Commun Biol. 2024 Jan 12;7:89. doi: 10.1038/s42003-024-05769-7 (PMC10786901; doi:10.1038/s42003-024-05769-7)
Supplement: Supplementary file 1 — Supplementary Information [file 42003_2024_5769_MOESM1_ESM.pdf]

Superior haplotypes of key drought-responsive genes reveal opportunities for the development of climate-resilient rice varieties

Preeti Singh[1,†], Krishna T Sundaram[1,†], Vishnu Prasanth Vinukonda[1,†], Challa Venkateshwarlu[1], Pronob J Paul[1], Bandana Pahi[1], Anoop Gurjar[2] Uma Maheshwar Singh[2], Sanjay Kalia[3], Arvind Kumar[2,4], Vikas K Singh[1,*], Pallavi Sinha[1,*]

[1]International Rice Research Institute (IRRI), South-Asia Hub, Hyderabad, India
[2]International Rice Research Institute, South Asia Regional Centre (ISARC), Varanasi, India
[3]Department of Biotechnology, CGO Complex, Lodhi Road, New Delhi, India
[4]Present Address: International Crops Research Institute for the Semi-Arid Tropics, Hyderabad, India

[†]These authors contributed equally

[*]Author for Correspondence: p.sinha@irri.org; v.k.singh@irri.org

Additional Information

The following Supplementary Material is available for this paper.

1. Supplementary Figures 1-6 (available in this file)
2. Supplementary Tables 1-7 (available in this file)
3. Supplementary Datasets 1-9 (available as MS Excel files)

Supplementary Table 1. Variance components due to genotypes ($\sigma^2 g$), mean, range, repeatability (R^2), and other parameters of single plant yield (SPY) across different seasons

**Trials**	**Conditions**	**Trait**	σ^2_g	**Mean**	**Range**	**GCV (%)**	R^2	**GAM (%)**
**2019 DS**	Normal	SPY	13.78^{**}	15.00	2.5-33.3	21.870	77.520	39.67
**2019 DS**	Stress	SPY	2.33^{**}	3.10	0.6-16.8	40.390	76.240	72.65
**2020 DS**	Normal	SPY	10.73^{**}	15.50	4 - 35.8	18.830	67.620	31.89
**2020 DS**	Stress	SPY	2.79^{**}	2.60	0.4-15.6	51.250	77.170	92.73

DS: Dry Season, GCV: Genotypic coefficient of variance, R^2: repeatability, GAM: Genetic advance of percent of means. **Significant at* $P \leq 0.01$

Supplementary Table 2. List of genotypes carrying superior haplotypes for seven drought-responsive genes

S.No.	Gene	*LOC_ Id*	SH	Variety	Geographic origin	Iris _id	Pop	SPY (g)	DSI
1	*OsDREB1C*	*LOC_Os06g03670*	H3	HODARAWALA	Sri Lanka	IRIS-313-10020	aus	6	0.84
				DONRADAO	Brazil	IRIS-313-11411	ind2	5.2	1.01
				MERLE	France	IRIS-313-8215	admix	3.4	0.98
				MULLIKURUVA	India	IRIS-313-9557	ind2	2.7	1.09
				CHANDINA	Sri Lanka	IRIS-313-9917	ind1B	8.9	0.56
2	*OsDIL1*	*LOC_Os10g05720*	H22	LUBANG PUTI	Philippines	IRIS-313-10513	admix	1.3	1.09
				T. KAUG PAO	Taiwan	IRIS-313-11157	ind1A	7.1	0.81
				NAM SA-GUI 19	Thailand	IRIS-313-8485	ind3	5.5	0.92
				KEERIPALA CHILL PADDY	India	IRIS-313-8559	ind2	3.5	1.10
				E ZI 96	China	IRIS-313-8859	ind1A	5.2	0.93
3	*DSM3*	*LOC_Os03g12840*	H4	L 10833	China	IRIS-313-11751	admix	2.6	1.10
				IR 28	Philippines	IRIS-313-7691	ind1B	5.1	0.93
				CHANDINA	Sri Lanka	IRIS-313-9917	ind1B	8.9	0.56
4	*ASR3*	*LOC_Os02g33820*	H88	AUS 359	Bangladesh	IRIS-313-11060	aus	5.2	0.81
				LAL TAURA	India	IRIS-313-11170	aus	7.5	0.75
				SADA AUS	India	IRIS-313-11173	aus	6.9	0.94
5	*ZFP182*	*LOC_Os06g03670*	H4	CAC75	India	IRIS-313-11354	ind2	1.7	0.92
				LINANGAN	Philippines	IRIS-313-11381	ind3	2.6	1.04
				SA MER	Thailand	IRIS-313-11392	ind3	1.8	1.10
				CHITRAKALI	India	IRIS-313-11408	ind2	4.1	0.99
				DONRADAO	Brazil	IRIS-313-11411	ind2	5.2	1.01

ARC 18533	India	IRIS-313-11443	admix	10.1	0.57
NAPDAI	India	IRIS-313-11446	ind2	5.8	0.83
TUNAGANNAPNANG	India	IRIS-313-11447	admix	4.9	0.91
KARAHANI	India	IRIS-313-11458	aus	3.2	0.72
SALSI	India	IRIS-313-11462	aus	7.8	0.67

Supplementary Table 3. List of potential donors for SPY under drought stress in rice

**Genotype**	**Population**	**Country**	***ZFP182***	***OsDREB1C***	***DSM3***	**SPY(g)**
DONRADAO	indica	Brazil	H4	H3		5.2
CHANDINA	indica	Sri Lanka		H4	H4	8.9

Supplementary Table 4. Sequencing details of the eight rice varieties and mapping of sequence reads

Sample	Total reads generated (Million reads)	Reads mapped (Million reads)	Genome coverage at 1X	Average depth (X)
DRR Dhan 42	121.40	119.24	94.07%	44.9
DRR Dhan 44	84.40	82.18	91.81%	31.0
DRR Dhan 46	142.90	139.49	93.24%	52.0
Sahbhagi Dhan	51.74	51.21	90.91%	16.2
Naveen	71.33	70.22	91.22%	26.1
Swarna	70.24	68.81	91.41%	25.8
BPT 5204	78.05	76.98	91.52%	29.0
DRR Dhan 48	72.54	70.80	91.49%	26.7

Note: Details of the sequencing data of these eight varieties are available under the following accession numbers: ERR12304009 (DRR Dhan 42), ERR12303753(DRR Dhan 44), ERR12305485 (DRR Dhan 46), ERR12305492(Naveen), ERR12305489 (Swarna), ERR12305486 (BPT 5204), ERR12305487 (DRR Dhan 48v) and ERR12305488 (Sahbhagi Dhan).

Supplementary Table 5. Comparison of identified MTAs with previously reported QTLs

SNP _Id	Position	PVE(%)	Gene	*Os_ID*	Previously reported QTLs	References
S1_35970278	35970278	2.2461534	*OsDSR2*	*Os01g0839200*	$qDTY_{1.1}$	Vikram et al., 2011
S1_42747553	42747553	2.4933631	*OsDREB1F*	*Os01g0968800*	downstream of $qDTY_{1.1}$	Yadav et al., 2019
S6_182471138	1388018	2.0979761	*OsDREB1C*	*Os06g0127100*	upstream of $qDTY_{6.3}$	Yadav et al., 2019
S8_264245022	22215494	2.1087565	*OsDERF1*	*Os08g0454000*	upstream of $qDTY_{8.1}$	Catolos et al., 2017
S9_286465239	15992689	2.2241742	*OsDSG1*	*Os09g0434200*	$qDTY_{9.1}$	Dixit et al., 2012

Supplementary Table 6. Haplo-pheno analysis of the additional genes identified in the LD region in c-GWAS study

MSU	Gene symbol	Total no.of haplotype	Superior Haplotype	Lines with SH	Gene Description
LOC_Os01g62190	*ZFP179*	NA	-	-	ZOS1-15 20- 20C2H2 20zinc 20finger 20protein 2C expressed
LOC_Os03g12790	*GS3.1|DG1|GFD1*	4	NA	-	MATE 20efflux 20family 20protein 2C 20putative 2C expressed
LOC_Os03g60430	*OsIDS1*	NA	-	-	AP2 20domain 20containing 20protein 2C 20expressed
LOC_Os03g60509	*gh1|OsCHI*	8	NA	-	expressed 20protein
LOC_Os03g60570	*ZFP15*	4	H3	6	ZOS3-22 20- 20C2H2 20zinc 20finger 20protein 2C expressed
LOC_Os03g60580	*OsADF3*	2	NA	-	actin-depolymerizing 20factor 2C 20putative 2C 20expressed
LOC_Os06g03520	*OsFLZ18*	12	NA	-	DUF581 20domain 20containing 20protein 2C 20expressed
LOC_Os06g03580	*OsBBI1*	2	NA	-	zinc 20RING 20finger 20protein 2C 20putative 2C 20expressed
LOC_Os06g03610	*RUPO*	3	H2	212	TKL_IRAK_CrRLK1L-1.13 20- 20The 20CrRLK1L-1 20subfamily 20has
LOC_Os06g03640	*OsBAG3*	3	NA	-	BAG 20domain 20containing 20protein 2C 20expressed
LOC_Os08g35210	*OsrbohE|Osrboh6*	5	NA	-	ferric 20reductase 2C 20putative 2C 20expressed
LOC_Os10g05750	*OsPRP3*	9	H14	6	POEI3 20- 20Pollen 20Ole 20e 20I 20allergen 20and 20extensin
LOC_Os12g38170	*OsOSM1*	6	NA	-	osmotin 2C 20putative 2C 20expressed
LOC_Os12g38210	*SPL11*	NA	-	-	spotted 20leaf 2011 2C 20putative 2C 20expressed
LOC_Os12g38290	*OsMT1g*	NA	-	-	metallothionein 2C 20putative 2C 20expressed
LOC_Os12g38300	*OsMT1d*	NA	-	-	metallothionein 2C 20putative 2C 20expressed

Supplementary Table 7. Pedigree information of the drought-tolerant lines used for validation

**Drought Tolerant (DT) Variety**	**Immediate Parents**
DRR Dhan 42 (IR77298-14-1-2)	IR64/ADAY SEL//3*IR64
DRR Dhan 44 (83376-B-B-130)	IR 71700-247-1-1-2/IR 77080-B-34-1-1
DRR Dhan 46 (IR IR83383-B-B)	IR72022-46-2-3-3-2/ IR57514-TMI-5-B-1-2
Sahbhagi Dhan (IR 74371-70-1-1-1-B-1)	IR 55419-4*2/WAY RAREM
PSBRc 82 (IR64683-87-2-2-3-3)	IR47761-27-1-3-6/IRRI 108
PSBRc 68 (IR 57515-PMI8-1-1-SRN-1-1)	IR43581-57-3-3-6/IR26940-20-3-3-1
Aus 299	Traditional

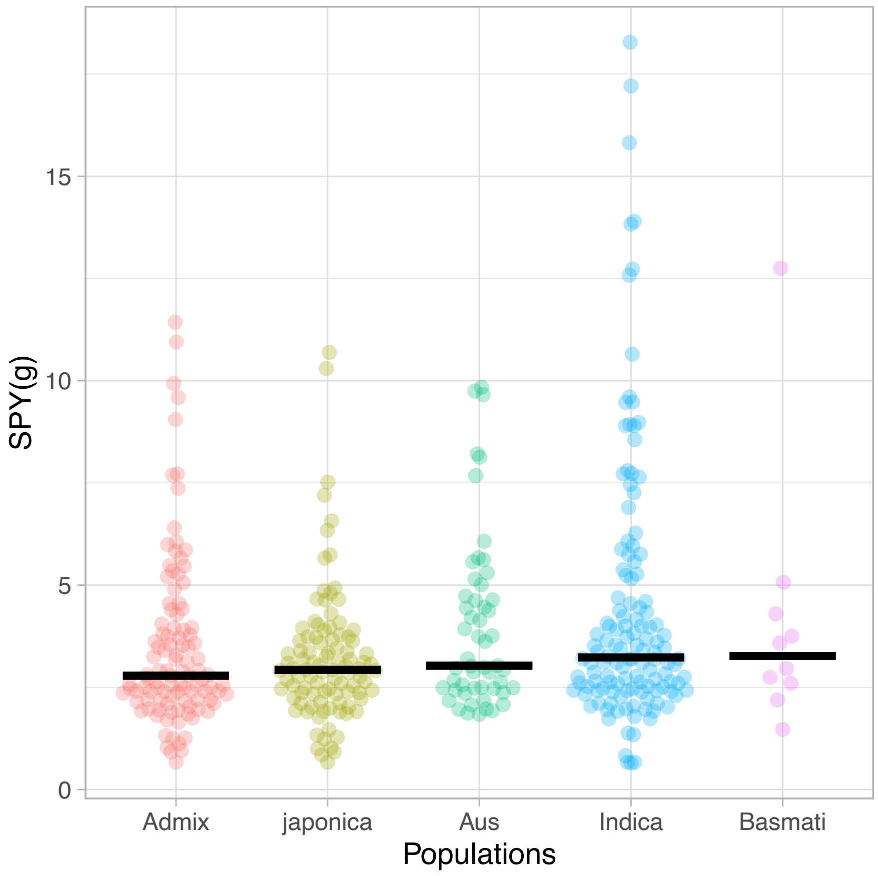

Supplementary Fig. 1. Phenotypic distribution of single plant yield (SPY) trait under reproductive stage drought conditions in a subset of 399 diverse accessions of rice. The violin plots show the phenotypic distribution of the subpopulations within 399 subset of the rice reference set for SPY. The shape of the distribution (skinny on each end and wide in the middle) indicates that the trait distribution is highly concentrated around the median except for the basmati population.

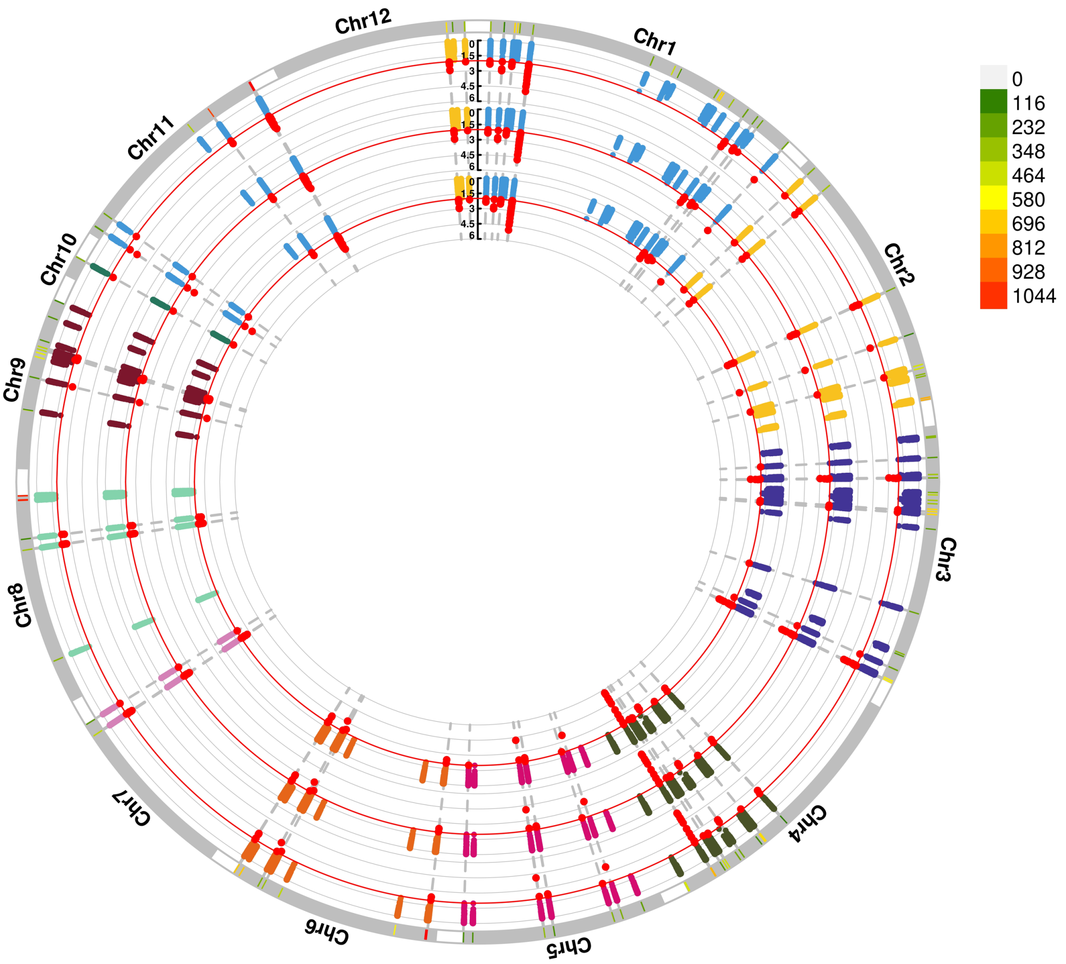

Supplementary Fig. 2. Circular Manhattan plots of c-GWAS for Single plant Yield (SPY) under reproductive stage drought stress using a panel of 399 genotypes. The inner circle to the outer circle represents the GWAS models used, MLMM, MLM and CMLM, respectively. Red dots present within the Manhattan circular plots indicate significant SNPs that exceeded the threshold, p-value = 0.005.

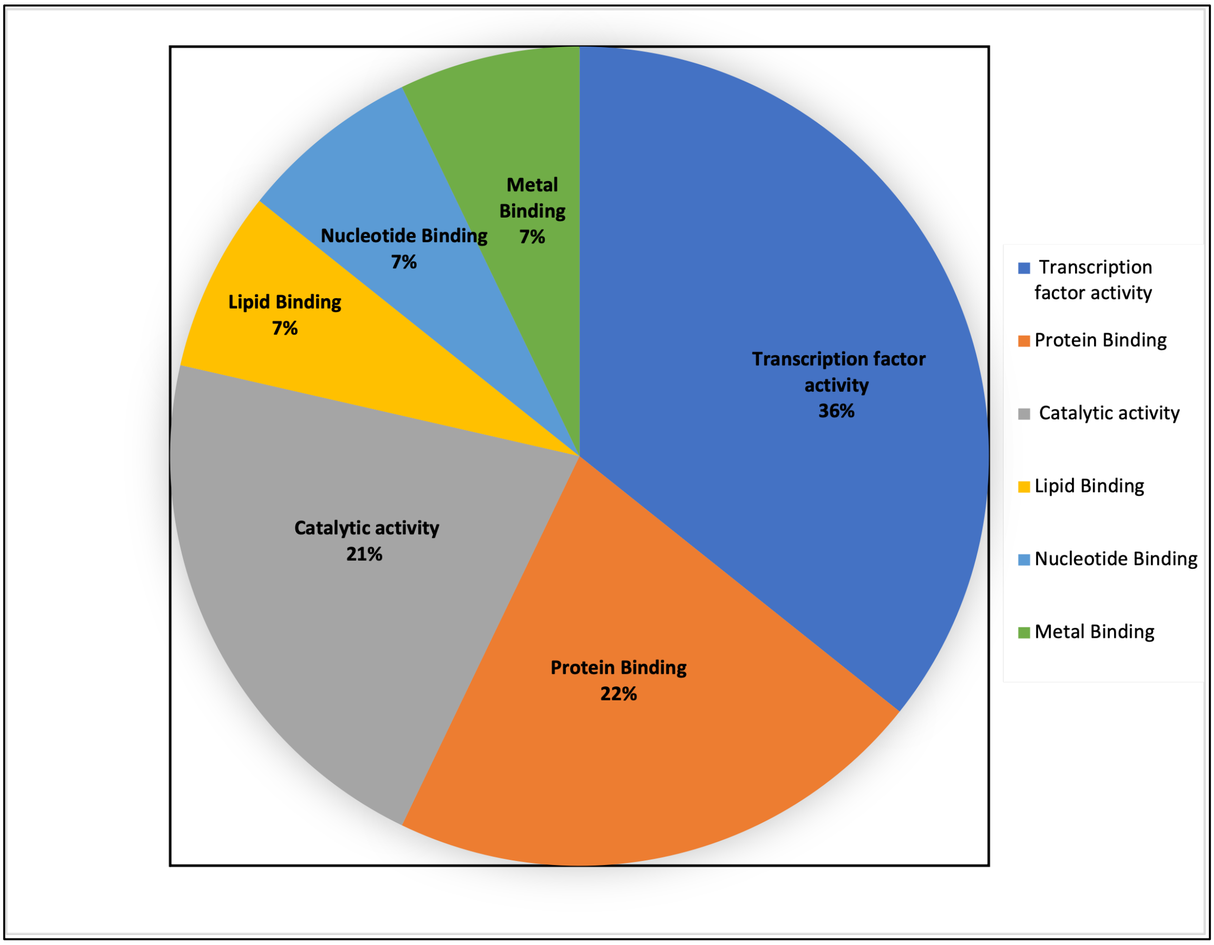

Supplementary Fig. 3. Pie chart describing Gene Ontology (GO) of molecular functions of the candidate genes for SPY.

A) B)

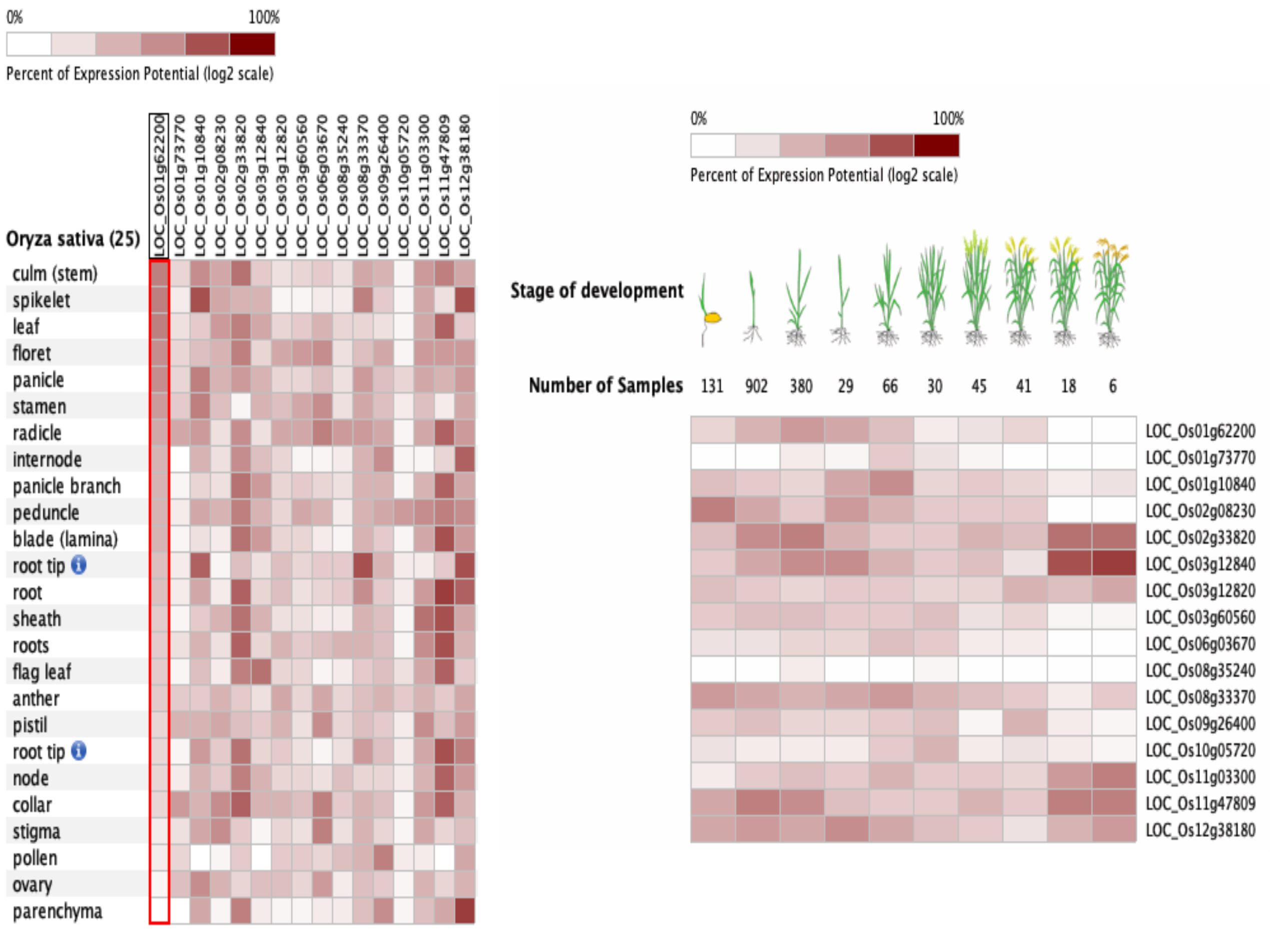

Supplementary Fig. 4. Expression analysis of the associated candidate genes in *Oryza sativa*. The heatmap was prepared using the Genvestigator tool. The dark and light colour shadings represent relatively high or low expression levels, respectively. **(A)** Expression analysis of candidate genes in different tissues. **(B)** Expression analysis of candidate genes at different life stages (germination, seedling, tillering, stem elongation stage, booting stage, heading stage, flowering stage, milk stage, dough stage and mature grain stage).

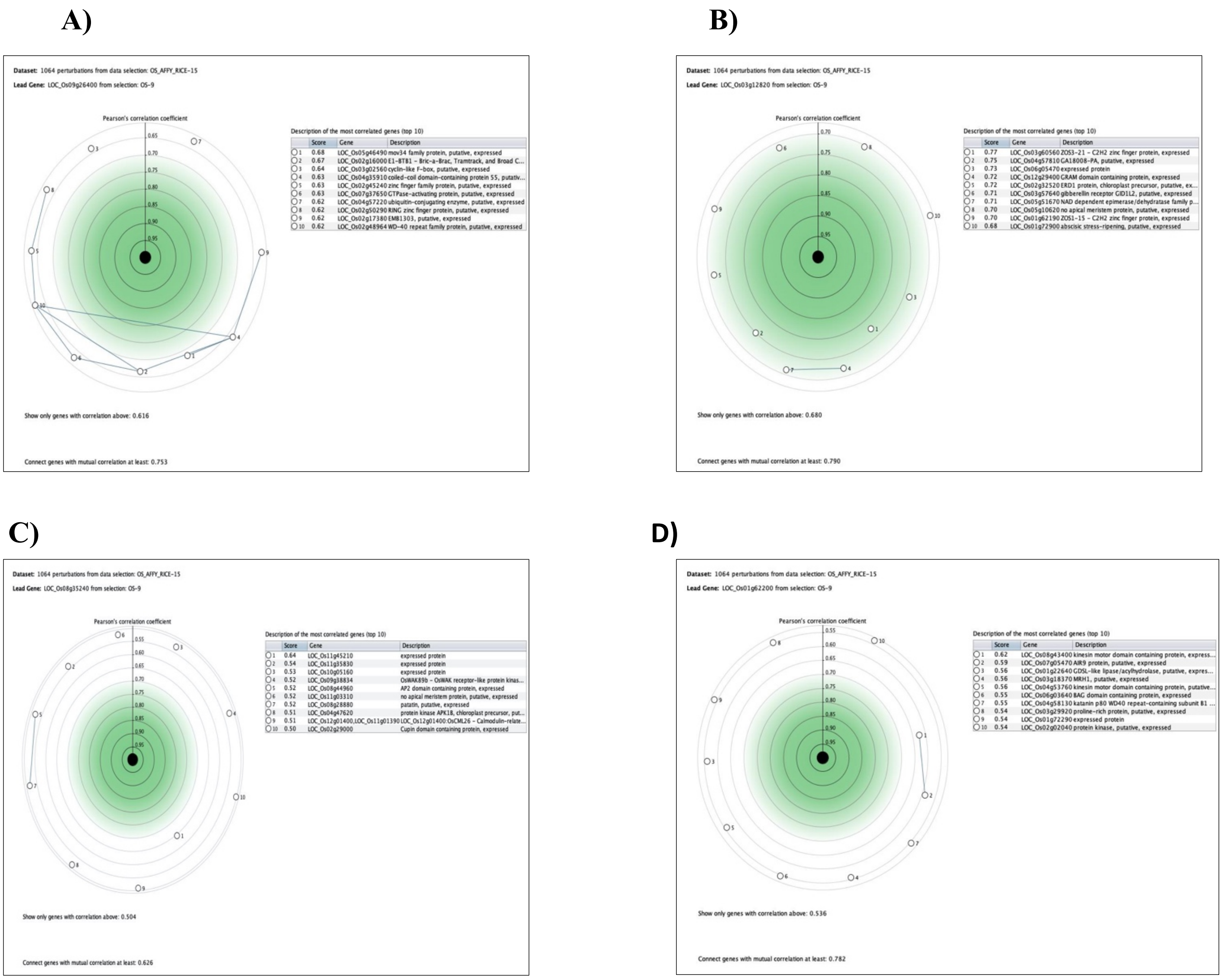

Supplementary Fig. 5. A co-expression network generated with the condition search tools and using the perturbations profile with Genevestigator tool. The top 10 positively co-expressed genes were displayed on a circular plot with Pearson correlation coefficient (PCC) score**. (A)** Co-expression profile of *OsDSG1* (**B)** Co-expression profile of *OsSRO1c* **(C)** Co-expression profile of *OsDERF1* **(D)** Co-expression profile of *OsDSR*2

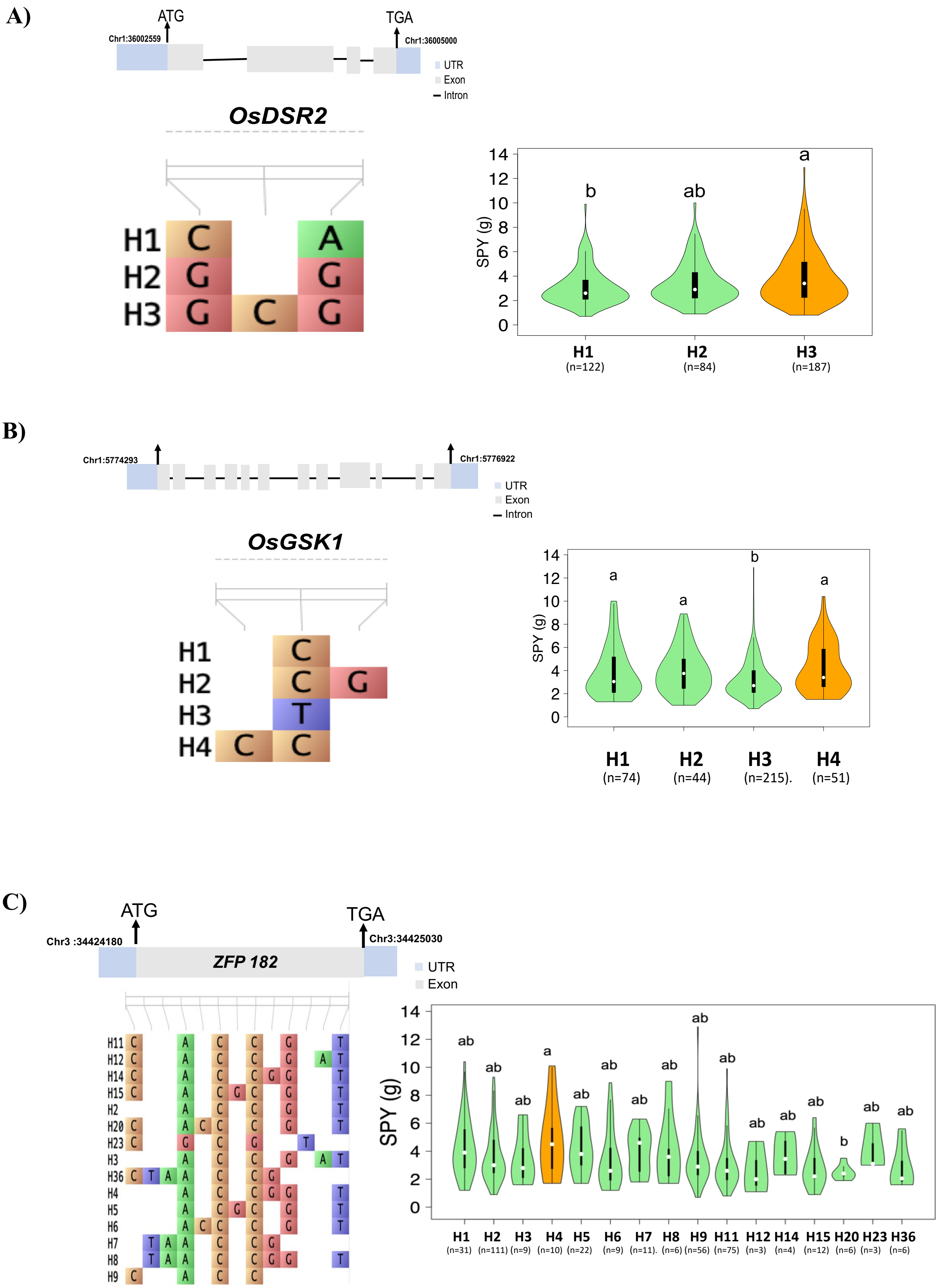

A)
ATG
TGA
Chr1:36002559
Chr1:36005000
UTR
Exon
Intron
OsDSR2
H1 C A
H2 G G
H3 G C G
SPY (g)
b
ab
a
H1 (n=122)
H2 (n=84)
H3 (n=187)
B)
Chr1:5774293
Chr1:5776922
UTR
Exon
Intron
OsGSK1
H1 C
H2 C G
H3 T
H4 C C
SPY (g)
a
a
b
a
H1 (n=74)
H2 (n=44)
H3 (n=215).
H4 (n=51)
C)
ATG
TGA
Chr3 :34424180
Chr3:34425030
ZFP 182
UTR
Exon
SPY (g)
H1 (n=31)
H2 (n=111)
H3 (n=9)
H4 (n=10)
H5 (n=22)
H6 (n=9)
H7 (n=11).
H8 (n=6)
H9 (n=56)
H11 (n=75)
H12 (n=3)
H14 (n=4)
H15 (n=12)
H20 (n=6)
H23 (n=3)
H36 (n=6)

D)

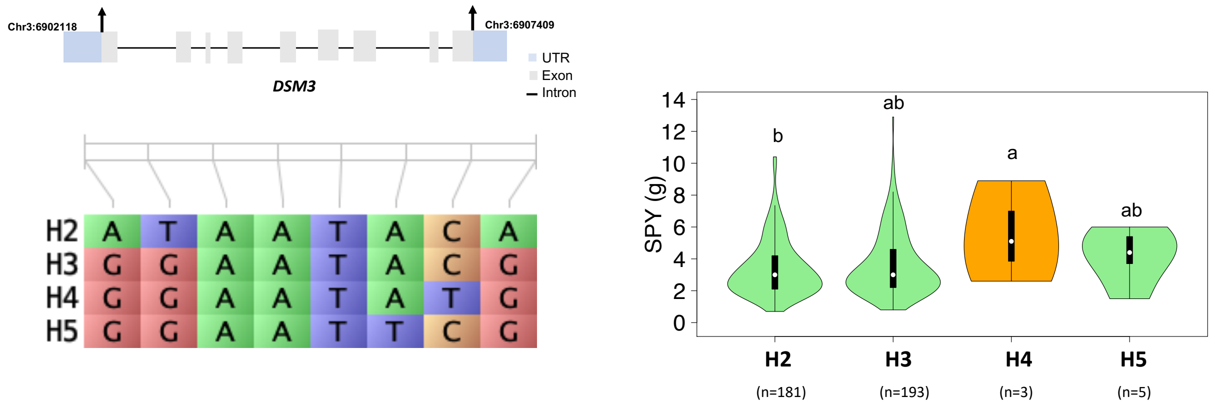

E

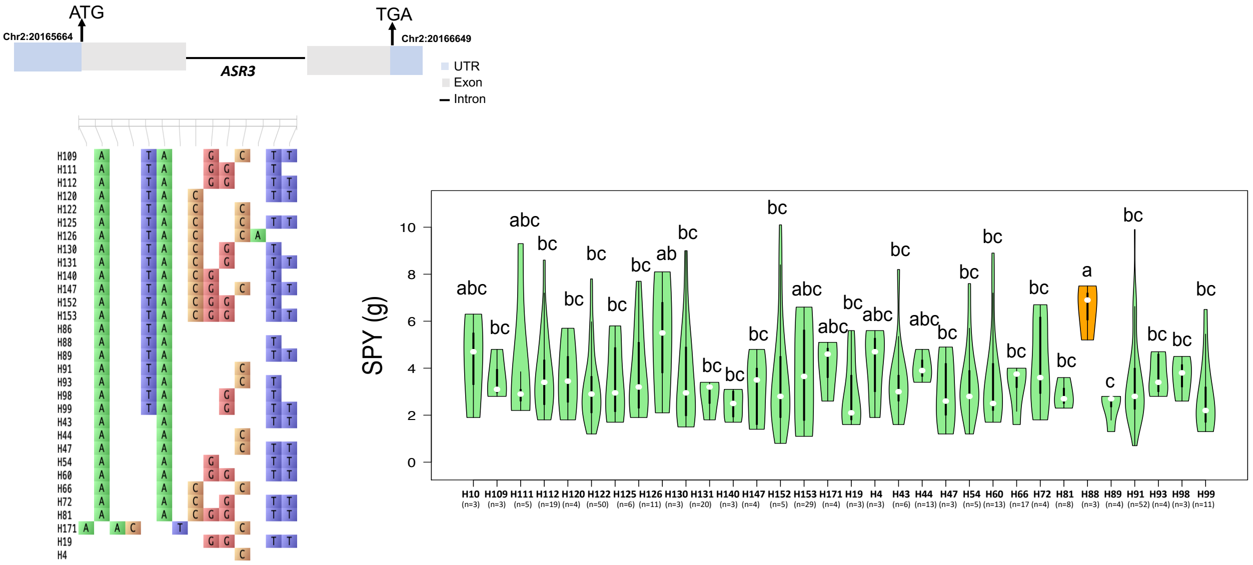

Supplementary Fig. 6. Haplotype analysis of the candidate genes associated with the trait across the subset panel. Haplotypic variation of *OsDSR2, OsGSK1, ZFP182, DSM3*, and *ASR3* gene associated with single plant yield trait (SPY) in the 3K-RG panel is shown in the left panel (**A-E**). Boxplot showing variation in SPY trait in rice accessions significant at p-value < 0.05 (right panel, **A-E**). Different alphabets denote significant differences between haplotypes and n=number of accessions carrying haplotypes. The violin plot uses an orange color to depict the distribution of the superior haplotype, while the green color is employed to represent the distribution of the remaining haplotypes associated with the gene.

SUPPLEMENTARY DATA (supplied as separate excel sheets)

Supplementary Data 1. Major functionally characterized genes governing drought tolerance in rice along with the number of haplotypes in the 3K-RG panel

Supplementary Data 2. Country-wise list of 3K-RG panel subset utilized for phenotyping the SPY trait in two seasons.

Supplementary Data 3. Candidate gene-based association analysis for identification of trait-associated genes

Supplementary Data 4: Haplotype distribution and frequency range of 16 selected genes in the established subset of the 3K-RG panel

Supplementary Data 5. Haplotype distribution and frequency range of 7 selected genes within different rice subpopulations in the established subset of the 3K-RG panel

Supplementary Data 6. In silico analysis of the SNPs substitution effects for the selected associated gene

Supplementary Data 7. List of KASP primers designed for the *OsDREB1C* gene

Supplementary Data 8. Candidate gene-based association analysis for identification of trait-associated genes with DSI

Supplementary Data 9: The source data behind the graphs in different figures in the paper.
