# Supplementary material for: Superior haplotypes of key drought-responsive genes reveal opportunities for the development of climate-resilient rice varieties
Source: Commun Biol. 2024 Jan 12;7:89. doi: 10.1038/s42003-024-05769-7 (PMC10786901; doi:10.1038/s42003-024-05769-7)
Supplement: Supplementary file 2 — Description of Additional Supplementary Files [file 42003_2024_5769_MOESM2_ESM.pdf]

Description of Additional Supplementary Files

File name: Supplementary Data 1
Description: Major functionally characterized genes governing drought tolerance in rice along with the number of haplotypes in the 3K-RG panel

File name: Supplementary Data 2
Description: Country-wise list of 3K-RG panel subset utilized for phenotyping the SPY trait in two seasons.

File name: Supplementary Data 3
Description: Candidate gene-based association analysis for identification of trait-associated genes

File name: Supplementary Data 4
Description: Haplotype distribution and frequency range of 16 selected genes in the established subset of the 3K-RG panel

File name: Supplementary Data 5
Description: Haplotype distribution and frequency range of 7 selected genes within different rice subpopulations in the established subset of the 3K-RG panel

File name: Supplementary Data 6
Description: In silico analysis of the SNPs substitution effects for the selected associated gene

File name: Supplementary Data 7
Description: List of KASP primers designed for the *OsDREB1C* gene

File name: Supplementary Data 8
Description: Candidate gene-based association analysis for identification of trait-associated genes with DSI

File name: Supplementary Data 9
Description: The source data behind the graphs in different figures in the paper.
